# Millicurrent stimulation of human articular chondrocytes cultivated in a collagen type-I gel and of human osteochondral explants

**DOI:** 10.1186/1472-6882-10-43

**Published:** 2010-08-06

**Authors:** Karsten Gavénis, Stefan Andereya, Bernhard Schmidt-Rohlfing, Ralf Mueller-Rath, Jiri Silny, Ulrich Schneider

**Affiliations:** 1 Aachen University Hospital, Dept of Orthopaedic Surgery, Pauwelsstr. 30, 52074 Aachen, Germany; 2 Aachen University Hospital, Dept of Trauma Surgery, Pauwelsstr. 30, 52074 Aachen, Germany; 3 Aachen University Hospital, Research Center for Bioelectromagnetic Interaction (femu), Pauwelsstr. 30, 52074 Aachen, Germany; 4 Arthro Nova Clinic, Wiesseerstr. 103, 83707 Ringsee, Germany

## Abstract

**Background:**

Here we investigate the effect of millicurrent treatment on human chondrocytes cultivated in a collagen gel matrix and on human osteochondral explants.

**Methods:**

Human chondrocytes from osteoarthritic knee joints were enzymatically released and transferred into a collagen type-I gel. Osteochondral explants and cell-seeded gel samples were cultivated in-vitro for three weeks. Samples of the verum groups were stimulated every two days by millicurrent treatment (3 mA, sinusoidal signal of 312 Hz amplitude modulated by two super-imposed signals of 0.28 Hz), while control samples remained unaffected. After recovery, collagen type-I, type-II, aggrecan, interleukin-1β, IL-6, TNFα and MMP13 were examined by immunohistochemistry and by real time PCR.

**Results:**

With regard to the immunostainings 3 D gel samples and osteochondral explants did not show any differences between treatment and control group. The expression of all investigated genes of the 3 D gel samples was elevated following millicurrent treatment. While osteochondral explant gene expression of col-I, col-II and Il-1β was nearly unaffected, aggrecan gene expression was elevated. Following millicurrent treatment, IL-6, TNFα, and MMP13 gene expression decreased. In general, the standard deviations of the gene expression data were high, resulting in rarely significant results.

**Conclusions:**

We conclude that millicurrent stimulation of human osteoarthritic chondrocytes cultivated in a 3 D collagen gel and of osteochondral explants directly influences cell metabolism.

## Background

Electrical stimulation for pain relief is a well established method in physical therapy centres. Mostly, it is combined with other treatments like massage, heat or physical manipulation. There are many commercial electrical stimulation devices available, which are commonly referred as transcutaneous electrical nerve stimulation units. These devices emit electrical pulses with alternating positive and negative polarities in the 10-500 kHz range and currents in the milliampere range. While units using higher currents are more effective in blocking acute pain, pain relief of units which deliver currents in the microampere range and frequencies from 0.5 to several hundred Hz can endure for several hours after end of treatment [1]. Polk et al investigated the beneficial effects of microcurrent treatment on soft tissue [2]. Clinically, diseases of the human locomotive system like pseudarthrosis have been treated with electromagnetic techniques since 1975 [3]. While some studies describe phenomenological effects of microcurrent treatment, the exact mechanism how microcurrent stimulation might affect chondrocytes in the hyaline cartilage environment remains unknown. When pressure is applied on hyaline cartilage, a change of electrical potentials can be observed which might induce intracellular changes in biosynthesis [4-6]. An enhancement of chondrogenic differentiation and of synthesis of cartilage extracellular matrix proteins has been described [7,8]. Additionally, the effect of microcurrent treatment on voltage-sensitive sodium and calcium ion channels is well documented [9]. One may speculate that these membrane-bound integrins may be involved in current signal transduction.

To our knowledge this is the first study which investigates the effect of millicurrent on human articular chondrocytes and on human osteochondral explants on the biochemical level. Nevertheless, the exact mode of action has to be elucidated in future studies.

## Methods

### Preparation of collagen gel seeded with human chondrocytes

Cartilage samples without any bone remnants were harvested from knee joints of 10 patients (2 male, 8 female; mean age 67.8) undergoing total knee replacement due to osteoarthritis. Only cartilage from morphologically unaffected regions from Outerbridge grade 3-4 patients [10] were included in the study. All patients gave their written consent prior to operation. The study was approved by the local ethics committee of the Aachen University Hospital.

Samples were collected in DMEM medium containing 10% fetal calf serum (FCS), 100 U/ml penicillin, and 100 μg/ml streptomycin. The cartilage was cut into 1-2 mm^3 ^pieces, and digested with 1 mg/ml Liberase 3 (Roche Diagnostics, Indianapolis, MN, USA) overnight. The released chondrocytes were washed subsequently for 3 times, and cell number was determined by CASY1 cell counter (Schärfe System, Reutlingen, Germany).

Rat tail collagen type-I gel was provided by Arthro Kinetics (Esslingen, Germany). The collagen type-I was supplied as an aqueous solution of 6 mg/ml in 0.1% acetic acid. It remained liquid when stored at 4°C and gelled when transferred to 37°C.

2 × 10^5 ^chondrocytes/ml gel were resuspended in 1 vol collagen type-I gel mixed with 1 vol 2× DMEM/2 M HEPES (0.93:0.07), resulting in a final concentration of 2 × 10^5 ^chondrocytes/ml gel. 1.5 ml cell-seeded collagen gel was given into each well of a 12-well-plate and allowed to gel for 30 min. After gelling, samples were overlaid with DMEM/FCS medium and cultivated under standardized in-vitro conditions (37°C, 5% CO_2_, humidified atmosphere) for up to 3 weeks. Every three days, samples were fed with fresh medium.

### Stimulation of cell-seeded collagen gel samples

Stimulation of collagen gel samples seeded with human chondrocytes was carried out by the Algonix device (Medilab, Würzburg, Germany).

**Figure 1:**
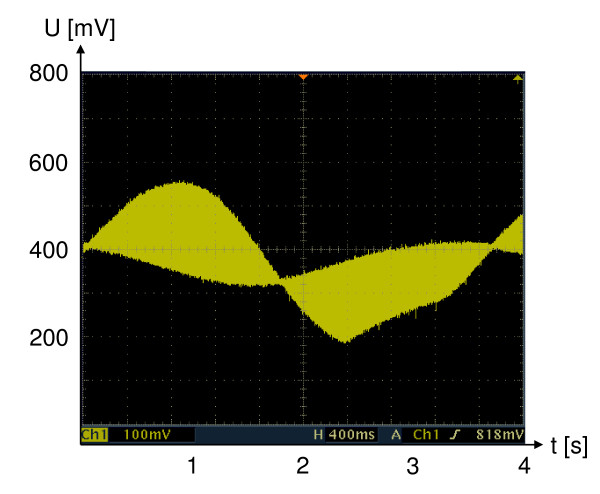
**Diagram of millicurrent stimulation**. Diagram of stimulation applied to cell-seeded collagen type-I gel samples and osteochondral explants. Electrodes of 3 mm in diameter were inserted at a distance of 3 cm. A current of 3 mA was applied; stimulation was carried out for 30 sec with a sinusiodal signal of 312 Hz amplitude modulated by two super-imposed signals of 0.28 Hz. Stimulation was repeated consecutively twice every two days for up to 3 weeks.

Sterile electrodes with a diameter of 3 mm were inserted at a distance of 3 cm on opposite sites into the cell-seeded gel samples. With a current of 3 mA stimulation was carried out twice for 30 sec consecutively with a sinusoidal signal of 312 Hz amplitude. The signal was modulated by 2 super-imposed signals with 2 different frequencies of 0.28 Hz (Fig. 1). By this, a current density of 10.6 A/cm^2 ^was achieved. Stimulation was repeated every two days. After up to 3 weeks, samples were recovered. One part of the specimens was processed for histological evaluation, while the remaining served for determination of gene expression.

The treatment group consisted of 10 independent samples of 10 patients. Ten samples of the same patients were left untreated and were cultivated in parallel as a control.

### Collection and stimulation of osteochondral explants

Human articular osteochondral samples consisting of cartilage and the underlying bone were harvested from knee joints of 10 patients (3 male, 7 female; mean age 67.2 years) undergoing total knee replacement due to osteoarthritis. Only specimens from morphologically unaffected regions from Outerbridge grade 3-4 patients was included in the study. All patients gave their written consent prior to operation. Samples were collected in DMEM medium containing 10% fetal calf serum (FCS), 100 U/ml penicillin, and 100 μg/ml streptomycin under sterile conditions.

Sterile electrodes with a diameter of 3 mm were pressed on opposite sites on the explants at a distance of 3 cm, while covered with medium. Every two days, stimulation was carried out as described and repeated twice. Osteochondral explants were cultivated under standard in-vitro conditions (37°C, 5% CO_2_, humidified atmosphere) for 3 weeks. As a control group, osteochondral explants from the same patients were left untreated and cultivated in parallel. After recovery, one part of the specimens was processed for histological evaluation. Cartilage from the remaining was depleted, and chondrocytes were released by enzymatic digestion as described. The released chondrocytes were subject to determination of gene expression.

### Histochemical and immunocytochemical analysis

Osteochondral explants were fixed and decalcified for 4 weeks and subsequently embedded in paraffin, while collagen gel samples were fixed overnight in a phosphate-buffered solution of 4% Para formaldehyde and embedded in paraffin. 5 μm sections were stained with hematoxylin-eosin and safranin O according to standard protocols.

For detection of collagen type-II protein, sections were deparaffinized, blocked and incubated with a polyclonal antibody to human collagen type-II (Biotrend, Cologne, Germany) diluted 1:50 overnight. Collagen type-I was detected by incubation of 1:200 diluted polyclonal antibody (Cedarlane, Hornby, Canada) overnight. Il-1β was visualized using a monoclonal antibody specific to human IL-1β (HyTest, Turku, Finland), while IL-6 was detected with a monoclonal antibody by the same manufacturer. TNFα protein was stained overnight with a polyclonal antibody 1:500 diluted by Abcam, Cambridge, USA. MMP-13 was detected by incubation with a 1:100 diluted monoclonal antibody (Chemicon, Temecula, CA, USA) at 4°C overnight.

Proliferating cells were detected by indirect immunohistochemistry using the Ki-67 polyclonal antibody (NeoMarkers, Fremont, CA, USA) staining an antigen specific for proliferating cells.

Apoptotic cells were detected by TUNEL staining of fragmented DNA (Dead End Colorimetric Apoptosis Detection System, Promega, Madison, WI, USA) according to the manufacturer's instruction.

Immunohistological staining was visualized using the streptavidin/biotin technique (Vectastain ABC Kit, Vector Laboratories, Burlingame, VT, USA). Diaminobenzidine (DAB Peroxidase Substrate Kit, Vector Laboratories, Burlingame, VT, USA) was used as the developing substrate leading to a brownish color of the immunopositive cells. The specificity of the staining was verified by omission of the primary antibody and the use of matrix samples without enclosed cells, giving the background staining.

All images were captured by a Leica microscope (Leica, Wetzlar, Germany) and prepared using the Discus software by the same manufacturer.

### Analysis of mRNA expression

RNA was isolated with the Oligotex Direct mRNA Kit (Quiagen, Hilden, Germany) according to the manufacturer's instruction. Isolated mRNA was used to create the corresponding cDNA by the SuperScript II First Strand Synthesis System (Invitrogen, Carlsbad, CA, USA). Gene expression of collagen type-II, collagen type-I, aggrecan, MMP-13, IL-6, Il-1β and TNFα was quantified by real time PCR on a LightCycler (Roche Diagnostics, Indianapolis, MN, USA). The housekeeping gene β-actin was used as an internal standard. Because of the large variability between the patients, data were presented as relative expression with respect to untreated control samples of the same patients. Primer sequences: Col-I (5-prime: GAGGGCCAAGACGAAGACATCC; 3-prime: CACAGAGGGAACCCAGGGAGC), Col-II (5-prime: CTGGTCCTTCTGGCCCTAGAG; 3-prime: AAAGGCGGACATGTCGATG), Aggrecan (5-prime: ATGCCCAAGACTACCAGTGG; 3-prime: TCCTGGAAGCTCTTCTCAGT) Il-1β (5-prime: TTTCCTGTTGTCTACACCAATGCC; 3-prime: GGGCTTTAAGTGAGTAGGAGAGG). TNFα and β-actin primer pairs were purchased from R&D Systems, Minneapolis, MN, USA. IL-6 and MMP-13 primer pairs were purchased from Maxim Biotech, San Francisco, CA, USA.

### Statistical analysis

For statistical evaluation the ANOVA test with repeated measurements was performed. An alpha level of 0.05 was chosen as the desired overall significance level.

## Results

### Stimulation of articular chondrocytes cultivated in a 3 D matrix

The collagen type-I gel was easily and homogeneously to mix with the freshly prepared chondrocytes. It could be poured into samples of different size, was easy to handle with a forceps and remained stable even when cultivated for 3 weeks in-vitro. Millicurrent stimulation did not lead to a remarkable improvement of the mechanical stability.

In all samples cells remained viable, as TUNEL staining revealed. Regardless of stimulation only few proliferating cells could be detected (data not shown).

**Figure 2:**
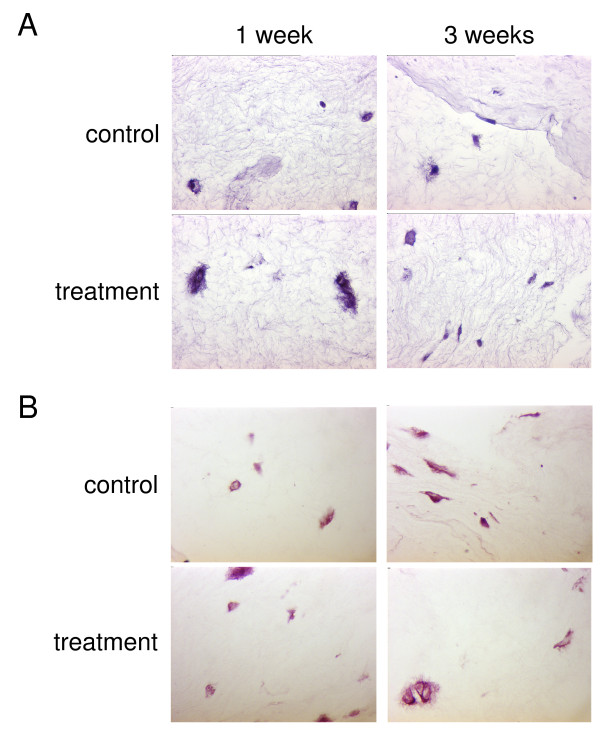
**H&E and safranin O staining of in-vitro cultivated specimens**. Hematoxylin-eosin staining (A) and safranin O staining (B) of collagen type-I gel seeded with human chondrocytes and cultivated for 1 and 3 weeks in-vitro. Samples were either left untreated (control) or stimulated by millicurrent treatment. Original magnification ×630.

In both control samples and samples stimulated by millicurrent treatment chondrocyte phenotype was prevailed to a large extent, although some cells showed signs of a partial morphological dedifferentiation (Fig. 2A). Proteoglycans were produced in both treatment and control specimens and stored mainly in the immediate cellular vicinity of the control group, while some cells of the treatment group started to build a matrix stretching out in the extracellular space (Fig. 2B).

**Figure 3:**
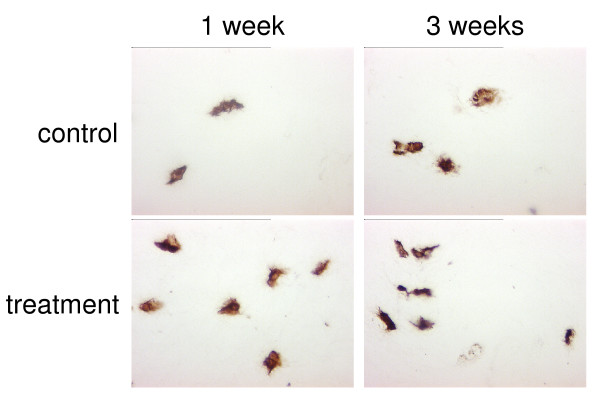
**Collagen type-II immunostaining of in-vitro cultivated specimens**. Immunohistological staining of collagen type-II protein in cell-seeded collagen gel samples left untreated or stimulated by millicurrent treatment after 1 and 3 weeks of in-vitro cultivation. Collagen type-II was detected mainly pericellularely. Original magnification ×630.

Collagen type-II protein was produced in both control samples and millicurrent-treated samples and stored mainly pericellularly. The building of a territorial matrix characteristic for hyaline cartilage could not be observed under the given in-vitro conditions (Fig. 3). The amount of collagen type-II protein as detected by semi-quantitative immunohistological staining did not reveal any obvious differences between control and millicurrent-treated samples, as did collagen type-I and proteoglycans (data not shown).

**Figure 4:**
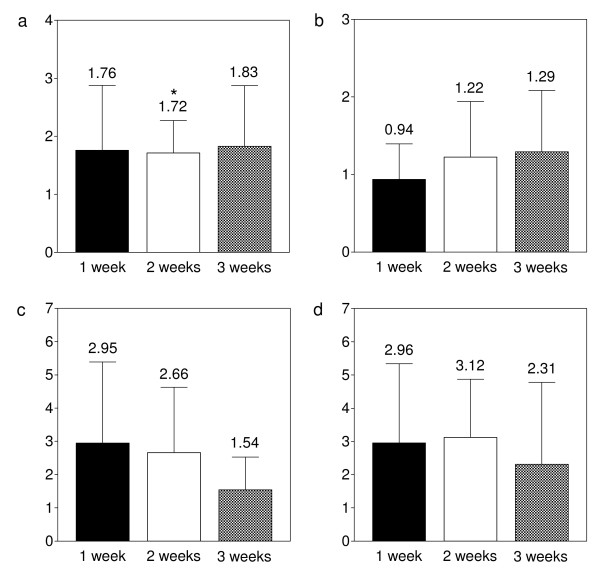
**Real-time PCR analysis of matrix-specific genes**. Real time PCR analysis of col-II (a), aggrecan (b), col-I (c) and interleukin-1β (d) gene expression. Data are given as relative gene expression of stimulated samples in relation to gene expression of untreated samples of the same patients, which were set as 1. B-actin gene expression was included as internal standard. Given are mean +/- SD. N = 10. *P *= 0.05.

When looking at the relative col-II gene expression levels of stimulated samples in relation to control samples of the same patients, millicurrent stimulation led to an increase of gene expression (Fig. 4). Even after 3 weeks of in-vitro cultivation, col-II gene expression was about 1.8-times elevated (*p *= 0.48 [1 week], *p *= 0.03 [2 weeks], *p *= 0.12 [3 weeks]). The increased col-II gene expression remained on a relatively constant level throughout the cultivation period. In contrast to col-II, there was no clear elevation of aggrecan gene expression detectable. After 3 weeks, aggrecan gene expression level was elevated to 1.3 (*p *= 0.45 [1 week], *p *= 0.35 [2 weeks], *p *= 0.34 [3 weeks]).

When looking at collagen type-I, gene expression was strongly elevated during the early cultivation period and was slowly decreasing while the cultivation proceeded. It was 4-times elevated after one week of cultivation; after 3 weeks, the elevation of col-I expression had decreased to 1.5 (*p *= 0.45 [1 week], *p *= 0.11 [2 weeks], *p *= 0.47 [3 weeks]).

With respect to the interleukin-1β gene expression, we found an 3-fold elevation for the treatment group after one week, which was decreasing to a 2.3-fold elevation after 3 weeks of cultivation (*p *= 0.16 [1 week], *p *= 0.16 [2 weeks], *p *= 0.38 [3 weeks].

### Stimulation of osteochondral explants

After in-vitro cultivation for 3 weeks, millicurrent stimulation did not lead to a macroscopic improvement of osteochondral explants. HE, safanin O and col-II staining did not reveal any obvious differences between control and treatment group, as did staining for IL-1β, IL-6, TNFα and MMP13 (data not shown). Additionally, TUNEL staining showed only single apoptotic cells in both groups (data not shown).

**Figure 5:**
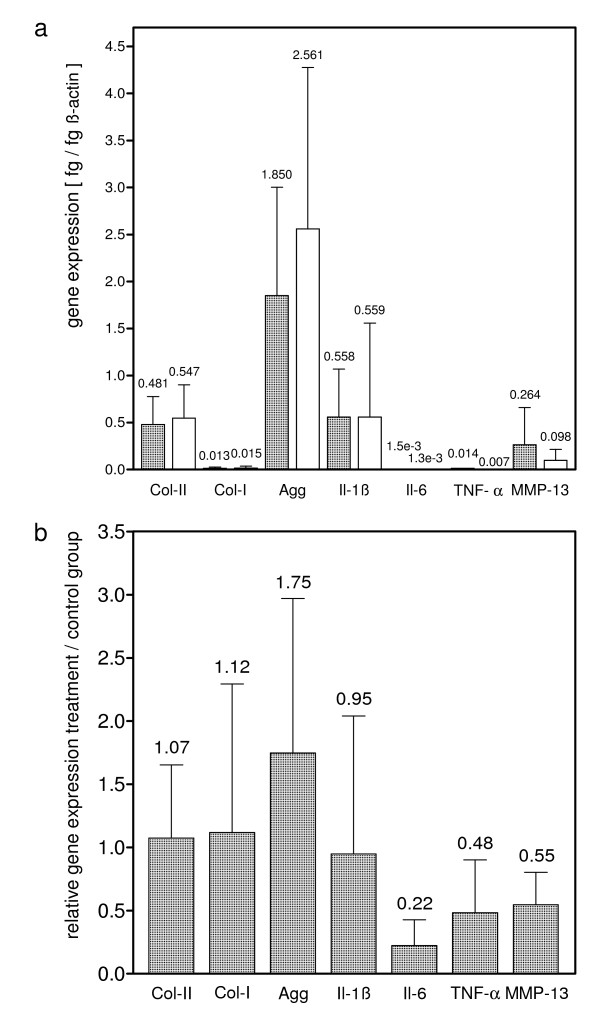
**Real-time PCR analysis of osteochondral explants**. (a) Gene expression of various genes of matrix synthesis, turnover and inflammation of osteochondral explants left untreated (left column) or stimulated by millicurrent treatment (right column). Specimens were cultivated in-vitro for 3 weeks. The housekeeping-gene β-actin was used as internal standard. *P *= 0.05. (b) Calculated relative gene expression of treatment samples in relation to control samples of the same patients, where gene expression of control samples was set as 1.

In contrast to semi-quantitative detection of protein production, determination of gene expression revealed some differences. The absolute and relative gene expression data are given in Fig. 5. While gene expression of collagen type-I and type-II was nearly unaffected, aggrecan gene expression was elevated by 1.75 fold (*p *= 0.45) in treatment samples with respect to control samples of the same patients. IL1β gene expression showed an equivalent level in both control and treatment group, while IL-6 gene expression in the treatment group decreased to 0.22 (*p *= 0.16). Treatment group MMP-13 gene expression decreased to 0.55 (*p *= 0.19), and TNFα decreased to 0.48 (*p *= 0.87) relative to control samples of the same patients.

## Discussion

Many efforts have been undertaken to improve matrix-based tissue engineering of hyaline cartilage, including manipulation of physical parameters like oxygen supply, improvements of matrix systems or stimulation with growth factors. Until now, only few studies have focused on the effects of stimulating articular chondrocytes with current treatment. Current stimulation of soft tissue for pain relief is a well established method since the 1980 [11-14]. Normally, it is combined with conventional methods like massage, heat or physical manipulation. While previous studies stimulated with current ranging from 20 μA to 600 μA [11,15,16], we applied a current of 3 Milliampere. This corresponds to a protocol which was successfully applied in pain treatment and post-operative rehabilitation by the manufacturer of the Algonix-device. Therefore, this slightly enhanced current was used in our study. Frank et al artificially damaged bovine articular cartilage by trypsin digestion and applied impedance analysis to the treatment site. They found that tissue impedance directly correlated with the degree of degradation [17].

To at least partly avoid cellular dedifferentiation, we chose to carry out the examinations on human chondrocytes embedded in a collagen type-I gel. Collagen gels are used as a matrix system for the cultivation of chondrocytes for many years and are of growing importance for tissue engineering of hyaline cartilage [18,19]. Additionally, osteochondral explants were subject for millicurrent stimulation.

Only little is known of the effects of current treatment of chondrocytes on the biochemical level. We found that cell metabolism of human chondrocytes is affected by current treatment in the milliampere range. We applied a current density of 10.6 mA/cm^2^, which should allow cellular stimulation. Investigating important genes of cartilage turnover, with respect to chondrocytes cultivated in a 3 D matrix in-vitro, we found a more universal up-regulation rather than a specific one. Aggrecan and collagen type-II gene expression, which is characteristic for hyaline cartilage, stayed elevated, while expression of the collagen type-I gene, indicating dedifferentiated chondrocytes, was slowly decreasing during cultivation. Immunohistological examination of protein production did not show any obvious enhancements with regard to col-II, col-I and proteoglycan production. This may be due to the relatively short cultivation period of 3 weeks. In contrast, when we treated osteochondral explants, we observed a specific pattern of gene expression, with some genes being stimulated (col-I, aggrecan), some genes being repressed (IL-6, MMP-13, TNF-α) and some left unaffected (col-II, IL-1β). One may speculate that the mechanisms of signal transduction are different with regard to 3 D gel culture and osteochondral explant culture, eventually because of missing ECM components in the collagen gel. On the other hand, we recently demonstrated a changed expression profile of a subset of integrins involved in signal transduction when we investigated chondrocytes with different states of differentiation [20]. Therefore, a different expression profile of signal-transducers of chondrocytes cultured in 3 D or explant culture may be involved.

Todd et al investigated the effects of electrical microcurrents generated by the ACE Stimulator on human dermal fibroblasts in-vitro [21]. They found that cell growth and viability were not influenced. When we were stimulating human articular chondrocytes with millicurrent, proliferation and apoptosis were unaffected, confirming these observations. They also observed an up-regulation of TGF-β1, which is an important regulator of cell-mediated inflammation and tissue regeneration. TGF-β1 secretion level was increased about 20-30%. We addressed the question of an inflammatory response to millicurrent therapy by monitoring the gene expression of Il-1β, IL-6 and TNFα.

Interleukin 1β plays a central role in the pathophysiology of cartilage damage and degradation in arthritis. It promotes the resolution system of cartilage matrix turnover through an increase in inflammatory cytokine and matrix metalloproteinase production by chondrocytes [22,23]. We found Il-1β gene expression to be elevated in chondrocytes cultivated in a 3 D matrix, while osteochondral explants did not show any differences.

When investigating Interleukin-6, Todd et al found no effect of microcurrent stimulation on dermal fibroblasts, while our results revealed a down-regulation following millicurrent treatment of osteochondral explants. IL-6 is not found in normal adult articular chondrocytes. It is strongly induced by the action of Il-1β [24]. In the presence of soluble IL-6 receptor, IL-6 has been shown to activate osteoclasts to induce bone resorption in vitro, suggesting that IL-6 may be involved in osteoporosis [25].

The proinflammatory cytokine tumor necrosis factor alpha (TNF-α) is not only critical for host defense against microbial agents but also plays an important role in joint inflammation and cartilage destruction in various forms of arthritis. TNF-α is expressed in the synovial lining cells and is present in the synovial fluid from patients with rheumatoid arthritis (RA) or osteoarthritis (OA) [26]. TNF-α is capable of activating the three subgroups of mitogen-activated protein (MAP) kinases in synovial fibroblasts and chondrocytes i.e. extracellular signal-regulated kinase (ERK), c-Jun Nterminal kinase (JNK), and p38 [27,28]. It has been reported that one or all of these MAP kinases are involved in regulation of IL-1β and MMP gene expression [29]. Regarding our results, TNFα gene expression was down-regulated following millicurrent treatment of osteochondral explant.

Additionally, we found gene expression of matrix metalloproteinase 13 (collagenase 3) to be down-regulated in osteochondral explants following millicurrent treatment. MMP13 levels are increased in cartilage and synovium of patients with arthritis [30-32]. Forsyth et al demonstrated that MMP13 expression is increased in aging human chondrocytes and could contribute to cartilage catabolism in osteoarthritis [33].

In summary, important genes of matrix degradation and inflammation are down-regulated in human osteochondral explants stimulated by millicurrent treatment in-vitro. Unfortunately, we found a high variation in gene expression between different donors, resulting in rarely significant gene expression data. Although we used cartilage tissue only from those patients with advanced osteoarthritis and undergoing total knee arthroplasty and from macroscopically unaffected areas, there was certainly some variability between the different donors. Therefore, strictly speaking, our results have to be interpreted as a strong indication rather than a proof. They have to be confirmed by further studies with a larger patient number.

In general, millicurrent therapy seems not only to suppress pain, but to directly influence cell metabolism in a selective way. This view is supported by our data. As no specific influence of millicurrent treatment on chondrocytes grown in 3 D gel culture could be demonstrated, the use in cartilage tissue engineering might be limited. The present work is limited to a phenomenological description of the millicurrent influence on chondrocyte metabolism. The underlying mechanisms have to be elucidated in future studies.

## Conclusion

We demonstrated that millicurrent therapy directly stimulates cell metabolism of human articular chondrocytes in human explants in a selective way. Therefore, millicurrent treatment may support regeneration of affected cartilage and may be a helpful tool to complement conventional therapy. Because of the demonstrated unspecific cellular stimulation with regard to 3 D gel culture, the benefits in cartilage tissue engineering might be limited.

## Competing interests

The authors declare that they have no competing interests. The Arthro Nova Clinic was not involved in the funding of this study.

## Authors' contributions

KG carried out the stimulation of the samples, the histological examinations and prepared the draft of the manuscript. BSR performed the statistical analysis. JS evaluated the millicurrent treatment protocol. US, SA and RMR participated in the study design and helped to draft the manuscript. All authors read and approved the final manuscript.

## Pre-publication history

The pre-publication history for this paper can be accessed here:

http://www.biomedcentral.com/1472-6882/10/43/prepub
